# AIRE's Complex Role Beyond Promiscuous Gene Expression

**DOI:** 10.1111/imr.70091

**Published:** 2025-12-29

**Authors:** Pärt Peterson

**Affiliations:** ^1^ Institute of Biomedicine and Translational Medicine University of Tartu Tartu Estonia

**Keywords:** autoimmune regulator, gene expression, thymus, type 1 interferons

## Abstract

Central tolerance, established in the thymus, ensures T cells are nonreactive to self‐antigens while maintaining a functional immune repertoire. Medullary thymic epithelial cells (mTECs) and thymic dendritic cells (DCs) play a crucial role in this mechanism by orchestrating the selection and deletion of autoreactive thymocytes, a process heavily influenced by the Autoimmune Regulator (AIRE). AIRE promotes the promiscuous expression of tissue‐restricted antigens (TRAs) in mTECs, enabling the elimination of self‐reactive T cells and the differentiation of regulatory T cells (Tregs). Studies on the role of AIRE in transcriptional regulation and nuclear body localization have significantly advanced our understanding of its mechanisms, revealing protein interactions with chromatin‐associated complexes that facilitate broad transcriptomic complexity in mTECs. However, the discovery of neutralizing type 1 interferon (T1 IFN) autoantibodies in APECED patients, alongside recently identified AIRE‐induced IFN signals in the thymus, suggests that AIRE's influence extends beyond its classical function in negative selection, potentially playing a broader role in thymic homeostasis via inflammatory signals. This review discusses recent advances in understanding AIRE's complexity, its role in transcriptional regulation and nuclear location, insights from studies in human APECED patients and rodent models, and the emerging concept of an IFN‐mediated tonic inflammatory signal in the thymus.

## 1 Introduction

During T cell development, thymocytes differentiate through a series of fate decisions to generate a functional yet self‐tolerant repertoire. This process depends on the spatially and functionally distinct compartments of the thymus: the cortex and medulla, where positive and negative selections predominantly occur, and Treg differentiation. In the thymic medulla, medullary thymic epithelial cells (mTECs), in coordination with thymic dendritic cells (DCs), are critical for exposing developing thymocytes to self‐antigens, ensuring tolerance to both ubiquitously expressed and tissue‐restricted antigens (TRAs).

The thymocyte selection begins in the cortex, where cortical thymic epithelial cells (cTECs) present a distinct repertoire of peptide–MHC complexes generated by specialized antigen‐processing pathways, including the thymoproteasome. Double‐positive (CD4+CD8+) thymocytes expressing TCRs with intermediate affinity for self‐peptide–MHC complexes are positively selected and survive to further differentiate into CD4+ or CD8+ single‐positive (SP) cells. Thymocytes with low affinity for these complexes undergo apoptosis through “death by neglect,” while those with excessively high affinity are directed toward deletion or Treg differentiation in the medulla.

The negative selection takes place in the medulla, where mTECs and thymic DCs present a broad array of self‐antigens, including TRAs. High‐affinity interactions between TCRs and these self‐antigens trigger apoptosis, eliminating autoreactive thymocytes and preventing autoimmunity. The mechanism of self‐antigen presentation is supported by promiscuous gene expression, regulated by the Autoimmune Regulator (AIRE) as it promotes the expression of thousands of TRAs in mTECs [1, 2]. Thymic DCs augment this process by capturing antigens from mTECs and presenting them to thymocytes, amplifying the diversity of antigens available for selection.

A subset of thymocytes encountering self‐antigens in the medulla is diverted into the regulatory T cell (Treg) lineage. These FOXP3+ Tregs play an indispensable role in maintaining immune tolerance by suppressing autoreactive T cells in the periphery. Their differentiation is driven by intermediate‐to‐high affinity interactions and durations between the TCR and self‐antigens within a defined affinity window that distinguishes Tregs from thymocytes undergoing deletion [3, 4].

This review discusses AIRE molecular functions, its interaction with chromatin, transcriptional mechanisms, and its location in nuclear body organization. Beyond this, the review highlights recent findings on AIRE's involvement in type 1 interferon (T1 IFN) signaling and suggests that AIRE's function extends beyond negative selection, playing a broader role in thymic homeostasis.

## 2 AIRE in Thymic Promiscuous Expression

mTECs are the primary source of TRAs within the thymus, and together with DCs, they provide the expression and presentation of antigens that are otherwise restricted to peripheral tissues. This mechanism extends tolerance to self‐antigens expressed in diverse organs throughout the body [5, 6]. Furthermore, mTECs efficiently express tumor antigens, which are either not expressed in normal cells or are expressed only at low levels. AIRE is central to this process, driving the transcription of thousands of TRAs and promoting their presentation of these antigens on MHC molecules [2, 7]. Notably, the high expression of AIRE is almost exclusively restricted to the mTEC^hi^ population. These cells are characterized by high levels of major histocompatibility complex (MHC) class II molecules and the CD80 co‐stimulatory marker [8, 9, 10], which are responsible for presenting TRAs on MHCII molecules to developing SP thymocytes.

The majority of TRAs are expressed in mTECs. mTECs have been estimated to express up to 85% of the coding genome, nearly 18,000–19,000 genes [11, 12]. In contrast, most tissues express 12,000–14,000 genes (approximately 60%–65% of the coding genome), with roughly 8000 protein‐coding genes expressed ubiquitously. The number of promiscuously expressed genes in mTECs is higher than that in two immunologically privileged tissues, brain and testis, known for their high transcriptional activity and expression in the range of 70%–75% of coding genes [13]. In Aire‐deficient mTECs, approximately 15,000 genes are expressed, indicating that Aire is responsible for the induction of approximately 3000–4000 genes in mTECs [11]. However, this suggests that of all the promiscuously expressed genes, only a minority (perhaps 20%–40%) are directly regulated by Aire. Although other regulators, such as Fezf2 [14] or Hipk2 [15] have been reported to contribute to promiscuous expression, the molecular drivers of the vast majority of TRA gene expression remain unknown.

Cell surface markers broadly classify mTECs into immature mTEC^low^ and mature mTEC^hi^ subsets, with the latter further divided into Aire‐expressing and, followed by terminal differentiation, into post‐Aire cells. The post‐Aire mTECs have low MHC class II and CD80 expression, and a proportion of these cells resemble skin keratinocytes expressing pemphigus‐related antigens such as desmogleins 1 and 3 [16]. Recent scRNAseq studies have revealed subsets of post‐Aire mTECs that mimic other peripheral cell types, suggesting an evolutionary mechanism that maximizes the thymus's capacity for self‐representation [17, 18, 19]. These cells acquire features of peripheral tissues, such as those of the gut, liver, and endocrine cells, thereby contributing to the antigenic diversity necessary for T cell differentiation.

The increased level of transcriptomic complexity in mTECs is not necessarily an innate property of each individual mTEC but rather a consequence of the overall transcriptomic diversity within the mimetic mTEC population. The higher level of transcription in the mTEC is reminiscent of that seen in the testis, where it results from different cell ensembles collectively expressing a broader range of genes [20]. While an individual mTEC can be transcriptionally narrow at a specific stage of differentiation, the overall mTEC population represents a wide range of transcriptomic complexity throughout thymic development. Indeed, it appears that only a small subset of mTECs express any given antigen. For example, insulin, a hallmark autoantigen of type 1 diabetes (T1D), is detected in only a small fraction of mTEC^hi^ cells at relatively low levels [10, 21], yet this expression is sufficient for negative selection as its depletion in mTECs leads to a breakdown of central tolerance and subsequent insulitis [22]. In the human thymus, insulin gene expression correlates with AIRE gene expression [23], and a VNTR polymorphism in the insulin gene, which is linked to the reduced thymic expression of insulin, has been associated with the development of T1D [24]. Studies in mouse models and the quantitative expression analyses in the human thymus have identified other cases where the deletion of a single antigen gene triggers autoimmunity [25, 26, 27]. However, the mTECs are not the sole source of TRAs in the thymus, as thymic B cells and DCs also contribute to the self‐antigen pool either by direct presentation [28, 29], or by cooperative antigen transfer with mTECs [30, 31].

The heterogeneous nature of mTECs introduces an additional layer of complexity to promiscuous expression. The identification of mimetic cells raises intriguing questions about their development and function, as evidence suggests they play a role in central tolerance and influence the homeostasis of other thymic‐resident populations [18, 32]. Mimetic cells are predominantly found in the post‐Aire mTEC population, and their numbers are significantly reduced in Aire‐deficient mice, suggesting that Aire is involved in their development [33]. However, they are not entirely absent in Aire‐deficient hosts, indicating that some mimetic cells can form independently of Aire, albeit in lower numbers. This observation raises the question of whether Aire's role in mimetic cell development is direct, for example, through its regulation of transcriptional networks or indirect, where its effects facilitate the emergence of these cell types via other upstream processes. It is likely that additional regulatory inputs, such as specific transcription factors, program cell type‐specific features in mimetic cells, suggesting that both Aire‐dependent and Aire‐independent mechanisms work together to shape the diversity and functionality of mTEC populations [33, 34].

In humans and other large mammals, epithelial structures known as Hassall's corpuscles (HCs) are found within the thymic medulla. These distinct, concentric clusters range from 20 to 100 μm in diameter and contain enucleated keratinized cells [35]. Their size correlates with overall thymic volume. In smaller rodents, such as mice, HCs are difficult to detect without antigen‐specific immunostaining [36]. In humans, the size and abundance of HCs appear to reflect thymic activity, as they increase during childhood and decline with thymic regression in adulthood. Notably, HCs are more abundant in cases of thymic hyperplasia, such as thymoma associated with myasthenia gravis [37]. Although relatively understudied, particularly due to their near‐invisibility in murine thymus samples, HCs likely represent the final differentiation stage of the post‐Aire mTEC lineage and can be stained using late epithelial markers such as cytokeratin CK10 and involucrin via immunohistochemistry [33, 38].

## 3 AIRE Enhances Transcriptional Activity

While AIRE is a pivotal driver of promiscuous gene expression in mTEC^hi^, it functions largely independently of the transcription factors that govern tissue‐specific expression of TRAs in peripheral cells. Unlike these factors, AIRE acts via a generic mechanism that does not rely on any particular proximal regulatory motif within the target gene. For example, when expressed in tissue culture cells, AIRE can amplify the expression of reporter constructs without defined promoter regions or mRNA processing elements [39]. Furthermore, genes activated by AIRE are influenced by the cellular environment in which they are expressed [40]. Thus, promiscuous expression in mTECs is not solely governed by AIRE's actions; rather, it functions as an amplifier of genes already poised for transcription within open chromatin regions opened in mTECs. The target genes are usually silent or expressed only at low levels and are transcribed in a stochastic manner, either monoallelically or biallelically, and using distinct alternative transcription start sites [41, 42].

Although promiscuous gene expression in mTEC^hi^ cells appears stochastic, it is also highly coordinated. The target genes tend to be derived from gene clusters that are located in relatively close genomic proximity [43, 44]. Single‐cell RNA‐seq studies have shown that mTECs express genes following an ordered pattern of stochasticity, meaning that individual mTECs express a somewhat random subset of TRA genes. The co‐expressed gene sets are either physically close on the chromosome or interact inter‐chromosomally but do not necessarily share other common characteristics, such as belonging to the same signaling or metabolic pathway [45, 46]. The co‐location in chromosomal clusters relates to AIRE's ability to bind superenhancers, enriched in topoisomerases TOP1 and TOP2 [47]. At these superenhancers, AIRE facilitates the formation of higher‐order chromatin structures, as demonstrated by high‐resolution chromosome‐conformation capture (Hi‐C) experiments conducted on ex vivo mTECs. AIRE was shown to enhance interactions between superenhancers and AIRE‐induced genes, whereas interactions with AIRE‐neutral genes were reduced. Interestingly, AIRE had a smaller impact on interactions between conventional enhancers and AIRE‐induced genes, indicating a preferential role in promoting interactions specifically between superenhancers and AIRE‐induced genes [47, 48]. By targeting superenhancers, AIRE facilitates the broad transcriptional activation of TRA genes. This is achieved through AIRE's ability to orchestrate chromatin looping via the cohesin complex, effectively bridging distant enhancers with their target promoters. Such three‐dimensional reorganization of chromatin architecture appears as a key function of AIRE as a potent transcriptional amplifier [48].

In addition to its direct interaction with superenhancer regions, AIRE is associated with other chromatin‐binding and transcriptional mechanisms. The conserved domains of AIRE primarily function as scaffolds for protein–protein interactions. This role has been highlighted by proteomic screens and small‐scale pull‐down experiments, which have identified dozens of AIRE interaction partners. These partners can be broadly classified into groups involved in chromatin binding, transcriptional regulation, and mRNA splicing [49, 50, 51].

Structurally, AIRE has a N‐terminal CARD region, a SAND (SP100, AIRE‐1, NucP41/75, DEAF‐1) domain, and two PHD‐type zinc fingers [52, 53]. With its first PHD1 zinc finger, AIRE interacts with repressive histone marks in chromatin [54, 55]. The electrostatically charged surface of the PHD1 domain provides a negative overall charge that enhances its binding to histone H3 unmethylated at lysine 4 (H3K4me0), a key marker of transcriptionally inactive genes in chromatin [54, 55]. The interaction with unmethylated histone H3 is, however, inherently low‐affinity and transient, indicative of a short‐term dynamic binding mechanism [54, 55]. AIRE‐PHD1 is exquisitely sensitive to histone H3 post‐translational modifications, and its fold disruption leads to the failure of the full‐length protein to bind to histone H3, which results in the consequent reduction of AIRE target genes activation [56]. By specifically targeting H3K4me0‐marked promoters, AIRE preferentially engages with transcriptionally silent loci, facilitating their activation. Nevertheless, this specificity is not absolute, as AIRE operates with a degree of stochasticity, allowing individual mTECs to express distinct subsets of TRAs. The PHD2 domain, while structurally similar to PHD1, features a positively charged surface, suggesting its involvement in distinct protein–protein interactions [50, 57].

Current evidence also supports a mechanism in which AIRE facilitates the release of paused RNA polymerase II (RNAP‐II) at silenced chromatin sites. This occurs through AIRE's recruitment of the positive transcription elongation factor b (P‐TEFb), which phosphorylates RNAP‐II pause factors and RNAP‐II itself, enabling the polymerase to transition into the elongation phase [51, 58, 59, 60]. Following transcriptional initiation, RNAP‐II is inhibited by negative elongation factors, causing elongation to stall near the promoter. The RNAPII activity is restored by P‐TEFb, which overcomes these negative regulators to enable productive elongation by RNAPII [61]. AIRE was shown to recruit P‐TEFb via its interaction with BRD4 [62]. BRD4 was reported to bind AIRE through its bromodomains, interacting with lysine residues in AIRE's CARD domain and acetylated by the histone acetyltransferase CBP. However, this acetylation‐dependent interaction was later disputed, demonstrating that BRD4 is dispensable for AIRE‐mediated transcription, as BRD4 inhibition or knockdown did not affect AIRE activity [63].

## 4 AIRE is Associated with DNA Break Complexes

Many of AIRE's binding partners are involved in the DNA damage response. Key partners include DNA topoisomerase 2‐alpha (TOP2A), DNA‐dependent protein kinase (DNA‐PK), its noncatalytic subunits Ku70/Ku80, and poly [ADP‐ribose] polymerase 1 (PARP1) [49, 50, 57, 64]. TOP2A relieves torsional stress in supercoiled DNA generated during transcription by introducing transient DNA breaks [65], which activate the DNA‐PK/Ku70/Ku80 complex. In turn, DNA‐PK phosphorylates nuclear proteins to initiate DNA repair [66]. DNA‐PK associated complex is also required for AIRE recruitment to chromatin and has been shown to phosphorylate AIRE at multiple serine and threonine residues [64, 67]. Although the kinase activity of DNA‐PK was not required for AIRE transactivation activity and the exact role of this phosphorylation in AIRE‐dependent gene activation remains unknown [67], mutation analysis of residues threonine 69 and serine 157 in the CARD domain—both potential DNA‐PK targets—indicates that phosphorylation may be a prerequisite for CBP‐dependent acetylation.

The interaction between AIRE and topoisomerases likely plays a key role in AIRE target gene expression, and both TOP1 and TOP2 have been identified as key AIRE‐interacting proteins [47, 49, 68]. Given that transcription induces topological perturbations, these interactions may facilitate a chromatin environment conducive to transcription [69]. As TOP1 and TOP2 are associated with DNA breaks, these are likely associated with the AIRE function.

For example, the induction of DNA breaks enhances the transcription of AIRE target genes [68]. Etoposide, a drug that stabilizes TOP2‐DNA complexes, was shown to enhance the expression of AIRE target genes and facilitate interaction between AIRE and TOP2 [49, 68]. Interestingly, low concentrations of camptothecin, a TOP1 inhibitor, also effectively stimulated AIRE‐dependent transcription. Like etoposide, camptothecin covalently traps the TOP1–DNA complex onto chromatin. The study by Guha et al. [68] further distinguished the functional effects of TOP1 and TOP2 inhibitors. While both etoposide and camptothecin induce DNA breaks and form covalent protein–DNA complexes, thereby promoting AIRE target gene expression, other inhibitors such as merbarone and β‐lapachone, which block TOP catalytic activity without generating DNA breaks, failed to enhance AIRE target gene expression. This suggests that both TOP1 and TOP2 inhibitors, which act by inducing DNA breaks, enhance AIRE target gene expression through similar mechanisms and transcriptional complexes. In addition, TOP2A knockdown resulted in a significantly reduced expression of AIRE target genes [49]. Single‐stranded DNA breaks may be the most significant contributor to AIRE‐dependent gene expression, as low‐dose etoposide primarily induces single‐stranded DNA lesions. The role of TOP1 in its interaction with AIRE was also identified through their co‐localization on superenhancers, supporting a model in which AIRE localizes to superenhancers to upregulate its target genes [47].

As highlighted earlier, the mTECs in the thymus and the spermatogonial stem cells in the testis are among the cell types with the highest transcriptional complexity. One consequence of high transcriptional activity is transcription‐associated mutagenesis and recombination, which leads to the accumulation of oxidative lesions, potentially serving as a source of mutagenesis within the cell's DNA [70, 71]. Although proofreading activities correct many such lesions during DNA replication, a minority may persist as a base pair mismatch in the double‐stranded DNA, forming a heteroduplex that recruits DNA repair machinery. Higher transcription also appears to form non‐B DNA structures such as hairpins, Z‐DNA, G‐quadruplexes, and RNA–DNA hybrids [70]. Furthermore, transcription‐induced supercoiling generates transient regions of single‐stranded DNA, which are chemically more reactive and susceptible to damage compared to duplex DNA. This aligns with recent findings demonstrating that AIRE‐induced gene expression is associated with Z‐DNA, a noncanonical, left‐handed double‐helical form of DNA [72]. ChIP‐seq analyses have shown an enrichment of Z‐DNA in chromatin peaks associated with AIRE and, conversely, an enrichment of AIRE in Z‐DNA peaks. Z‐DNA motifs were positively associated with the double‐strand breaks that correlated positively with poised promoters of AIRE‐inducible genes. According to this model, Z‐DNA may enhance the generation of DNA breaks at the promoters of AIRE target genes, thereby facilitating the maintenance of their poised chromatin status, enabling AIRE recruitment, and ultimately promoting gene expression [72]. Thus, AIRE's ability to drive promiscuous gene expression and high transcriptional activity in mTECs is associated with an increased incidence of DNA breaks and the presence of Z‐DNA structures at AIRE target gene promoters.

## 5 AIRE in Nuclear Structures

The N‐terminal CARD enables oligomerization, similar to the CARD domains in other proteins [73]. The oligomerization is needed for AIRE subcellular localization within nuclear bodies. For nuclear transport, AIRE also possesses a bipartite nuclear localization signal, which is located near the CARD domain [74, 75]. AIRE shares structural similarities with members of the Sp100 protein family within its CARD, SAND domains, and its first PHD finger [76, 77, 78]. Unlike AIRE, SP100 family members contain a bromodomain instead of a second PHD finger. This structural resemblance to AIRE highlights intriguing parallels, as Sp100 family proteins are chromatin regulators in immune cells. However, in contrast to promoting gene expression, their activity is mainly associated with silencing [79]. The Sp110 and Sp140 proteins also localize to nuclear bodies; however, these are distinct promyelocytic leukemia protein (PML) nuclear bodies. How SP100 family proteins differ from AIRE in their nuclear localization, chromatin associations, and transcriptional activity remains an open question.

Studies on AIRE mutations that disrupt the CARD domain have concluded that AIRE transcriptional activity depends on the formation of large macromolecular protein complexes. Structurally, the CARD domain adopts an ordered protein fold composed of six α‐helices, which assemble into a globular domain [73]. Furthermore, the domain shows the polarized distribution of electrostatic surface charges characteristic of other CARD domains responsible for homotypic protein–protein interactions. Mutations found in human patients partially or entirely disrupt the CARD domain's structure, leading to its unfolding and loss of function as a protein interaction module.

Many mutations affecting the CARD domain or other structural regions disrupt AIRE localization to nuclear bodies [73, 75, 80, 81, 82, 83, 84, 85, 86, 87, 88]. The nuclear bodies formed by AIRE resemble but do not entirely overlap with PML nuclear bodies [75, 89]. Importantly, these nuclear body structures are also present in mTECs [8]. Although the AIRE localization does not directly match PML bodies in the nucleus, the nuclear structures formed by AIRE and PML are often close and even share distinct proteins, suggesting structural and functional interactions. For example, AIRE is acetylated by histone acetyltransferases, CBP [82] and p300 [90], which are located in PML bodies. However, when overexpressed in the same cells, their location overlaps with AIRE nuclear bodies leading to enhanced AIRE‐mediated transcriptional activation [73, 81]. In contrast, artificially directing AIRE to PML bodies inhibits its transcriptional activity. Also, dominant negative AIRE mutations result in its mislocalization to PML bodies [91]. As PML bodies are known sites of transcriptional repression, this mislocalization effectively suppresses AIRE's function [92]. Additionally, the AIRE interaction with another PML nuclear body protein, DAXX, was identified by coimmunoprecipitation experiments [93]. This study also showed that DAXX exerts a strong repressive role on the transcriptional activity of AIRE. Thus, the aberrant sequestration of AIRE in alternative nuclear structures, such as PML bodies, leads to a loss of function and inhibition of its transcriptional activity.

Whether AIRE nuclear bodies serve as active sites of transcription has been a subject of debate. Early studies found that, similar to PML nuclear bodies, the core of AIRE nuclear bodies is devoid of chromatin and RNAP II, suggesting that these structures do not directly participate in transcription [81]. In contrast, recent findings indicate that AIRE transcriptional activity occurs within its nuclear structures, which form at CBP/p300‐rich enhancers to drive gene activation [81, 91]. Together, these studies demonstrate that precise subnuclear localization and interactions with partner proteins modulate its transcriptional activity. Nevertheless, as the AIRE nuclear bodies are bound to the nuclear matrix, they may influence transcriptional processes indirectly by reshaping the chromatin landscape or by facilitating the formation of transcriptional hubs [89, 94].

## 6 Parallels with PML Nuclear Bodies

Studies on PML nuclear bodies have shown that their dynamic nature influences chromatin organization and status, potentially altering gene expression profiles in response to cellular states and stress conditions [95]. Like AIRE‐positive nuclear dots, PML bodies range in size from 0.2 to 1 μm in diameter and are membrane‐less structures organized in a core‐shell manner via liquid phase separation. They function as ‘assembling hubs’ that modulate chromatin architecture and gene expression, with evidence suggesting their role in regulating adjacent chromatin under cellular stress conditions. Core PML nuclear body proteins include PML, DAXX, ATRX, and Sp100 [96]. For nuclear body formation, PML acts as a scaffold forming disulfide‐crosslinked covalent multimers that self‐organize into the body's outer shell. This outer shell then serves as a platform for recruiting partner proteins, such as the nuclear antigen SP100, which forms the inner shell, and DAXX, which constitutes the core of the structure.

PML bodies have been extensively studied in the context of herpesvirus infection, where they play a role in T1 IFN signaling. The genomes of herpesviruses such as HSV‐1 or CMV become associated with PML bodies upon nuclear entry, leading to the epigenetic silencing of viral genomes. Moreover, the expression of the PML gene itself is induced by T1 IFNs, and PML is required for the transcription of interferon‐stimulated genes (ISGs) following T1 IFN stimulation. This activation results in an increase in the size and number of PML nuclear bodies, which become juxtaposed to ISG loci during prolonged T1 IFN treatment [97]. IFN‐α has been shown to enhance global cellular SUMOylation in a PML‐dependent manner [98], leading to increased IFN‐α‐induced protein ubiquitination and ISGylation—the covalent attachment of ISG15 to lysine residues on newly synthesized proteins in a ubiquitin‐like fashion. This modification in turn influences the stability of several ISG products [98, 99]. Additionally, IFN‐dependent PML SUMOylation promotes the recruitment of DAXX to PML bodies [100].

However, PML is not merely a downstream target of the IFN pathway as it also functions as a positive regulator of IFN signaling. Specifically, PML enhances both IFN synthesis and the IFN‐induced expression of ISGs [92]. Furthermore, PML has been shown to associate with transcription factor complexes involved in the regulation of IFN and ISG expression, stabilizing their components and increasing promoter occupancy; beyond IFN signaling, PML also regulates the expression of other cytokines [101, 102].

Intriguingly, PML bodies appear to be interconnected with the DNA break repair pathway and the DNA sensor IFI16, which activates the STING–T1 IFN pathway during herpesvirus infections [103]. Following the initiation of DNA break repair, the number of PML bodies per cell increases, and PML is located to sites of DNA damage. Additionally, IFI16 has been shown to associate with PML bodies, further supporting the role of PML in DNA damage responses and antiviral immunity [104].

## 7 Lessons from Human Disease

Research in mouse models has highlighted the role of Aire in T cell differentiation, offering insights into the disease pathology in human AIRE‐deficient patients with autoimmune‐polyendocrinopathy‐candidiasis ectodermal dystrophy (APECED) [105, 106]. In Aire‐deficient mice, defective negative selection of thymic CD4+ T cells leads to autoreactive T cells in the periphery [107, 108, 109]. Furthermore, Aire deficiency is associated with reduced thymic Treg production, suppressing peripheral autoreactive responses [110, 111, 112]. Mice lacking Aire and Foxp3 genes develop severe autoimmunity early in life, exhibiting a more pronounced disease phenotype than either Aire‐ or Foxp3‐deficient mice alone [113]. Aire appears to influence the generation of a specific subset of Tregs, which may play a critical role in preventing autoimmunity. For example, the Treg defect in Aire‐deficient mice was specific to the thymic subset generated during the perinatal developmental stage, affecting only a portion of the thymic Treg repertoire [114]. Studies by Malchow et al. revealed that while most Treg specificities were generated independently of Aire, a small subset is Aire‐dependent, potentially contributing to the autoimmune manifestations observed in Aire knockout mice [111].

In APECED patients, a lower number of FOXP3+ Tregs, impaired Treg functionality, or reduced FOXP3 expression have been observed [115, 116, 117]. Additionally, AIRE‐deficient patient Tregs lacked common TCRβ clones, which were found instead in their conventional T cell compartment [118]. This finding suggested gaps in the Treg TCR repertoire of these patients. However, identifying the primary defects in APECED Tregs from peripheral blood samples has been challenging due to the high proportion of peripheral Tregs or activated CD25+ T cells, which obscure the contributions of thymic Tregs. Moreover, not all studies have found changes in the patient Tregs. For example, a study on naïve regulatory T cells in APECED patients found minimal overlap with helper T cell subsets, with clonal patterns similar between patients and controls [119]. Likewise, a recent study that replicated findings of decreased Treg numbers did not detect intrinsic defects in Treg functionality or significant differences in the TCR repertoire of expanded Tregs in APECED patients. However, some individual patients exhibited a more restricted usage of specific clonotypes [120].

Clinically, APECED is characterized as a monogenic condition predisposing to multiple endocrine and non‐endocrine autoimmune disorders [121, 122, 123, 124]. Although patients may develop multiple autoimmune diseases, three main features: autoimmune adrenocortical insufficiency (Addison's disease, AD), hypoparathyroidism (HP), and chronic mucocutaneous candidiasis (CMC) are by far the most common [121, 122]. Diagnosis is typically established when a patient presents with at least two of the three conditions. However, in cases where a sibling has a confirmed APECED diagnosis, the presence of just one of these components may be sufficient for diagnosis [124]. Beyond this classic triad, APECED is associated with a broader spectrum of less frequent disease manifestations, including additional autoimmune conditions and ectodermal anomalies. For instance, approximately 15%–20% of patients develop T1D [124]. Skin disorders, such as vitiligo, alopecia, and early‐onset urticarial rash, are also frequently observed. Notably, the severity and combination of clinical symptoms can vary widely among individuals, even among those with the same mutation or within the same population [122, 124, 125]. Interestingly, some autoimmune manifestations are reported more frequently in North American than in European patients [126, 127].

APECED typically begins in early childhood, with its features progressively developing over time. While the full triad is often complete by the second decade of life, some diseases may occasionally present later. Recent longitudinal study showed that most disease components appeared before age 15, and the number of clinical manifestations increased with age, showing a rapid rise until age 25 [128]. The number and severity of the clinical symptoms vary greatly between populations and even among patients with identical mutations [122, 124, 125]. It is noteworthy that APECED patients rarely develop certain well‐known autoimmune or autoinflammatory diseases, such as lupus, rheumatoid arthritis, multiple sclerosis, or psoriasis [129].

## 8 Autoantibodies to Autoantigens Expressed in Specialized Tissues

The hallmark of APECED is the presence of high‐titer autoantibodies targeting a wide range of autoantigens, of which some are expressed in specific tissues. Several prevalent autoantibodies are associated with endocrine tissues. For example, Addison's disease in APECED is linked to autoantibodies against steroidogenic P450 cytochrome enzymes, including steroid 21‐hydroxylase (P450c21 or CYP21), expressed in the adrenal cortex, as well as steroid 17‐alpha‐hydroxylase (P450c17 or CYP17) and the side‐chain cleavage enzyme (P450scc or CYP11A1), both of which are expressed in the adrenal glands and gonads [130, 131, 132, 133, 134]. Autoantibodies against glutamic acid decarboxylases (GAD1 and GAD2), characteristic of T1D, and against thyroid peroxidase (TPO) and thyroglobulin, which are frequently present in APECED patients with autoimmune thyroid disease, are also prominent [135, 136]. Additional common autoantibodies target aromatic L‐amino acid decarboxylase (AADC), tryptophan hydroxylase (TPH1), tyrosine hydroxylase (TH), and leucine‐rich‐repeat protein 5 (NALP5) [136, 137, 138, 139]. NALP5 is predominantly expressed in the cytoplasm of parathyroid chief cells and has been associated with hypoparathyroidism in APECED [140].

Systematic analyses of autoantibodies in APECED provided more insights into their targets. While most APECED patients displayed autoreactivity toward approximately 100 self‐proteins, shared among patients, there is a significant inter‐individual variation in autoantigen profiles, collectively encompassing hundreds of autoantigens [141]. Interestingly, APECED autoantibodies target cancer‐testis antigens, especially associated with melanoma, including members of the MAGE‐A, MAGE‐B, and GAGE families, and sperm‐specific proteins, suggesting a role for AIRE in antitumor immunity with potential implications for cancer immunotherapy [142]. However, only approximately 10% of the targeted autoantigens had a tissue‐restricted expression pattern [142]. The autoantigens targeted in APECED patients appear to be proteins expressed not only in mTECs but also in lymphoid cells [142]. The presence of autoantibodies varies widely among patients, with many detected in only a limited number of individuals [141]. Developing a unique antibody repertoire may depend on genetic factors or reflect an indirect response to tissue destruction, independent of direct pathogenesis. Notably, many autoantigens are intracellular, and while some, such as steroidogenic enzymes, are tissue‐specific, others are expressed ubiquitously. Interestingly, many identified human autoantigens, which are frequently recognized by APECED patient autoantibodies, are not expressed in an AIRE‐dependent manner [143].

## 9 Autoantibodies to T1 IFNs in APECED are Shared with Thymoma Patients

A particularly intriguing feature of APECED is the presence of autoantibodies targeting cytokines, which modulate immune responses. Among the most significant are high‐titer autoantibodies against T1 IFNs, specifically IFN‐α and IFN‐ω, which are detected in nearly 100% of patients [144]. IFN‐β is targeted less frequently with a prevalence of approximately 5%–10% of cases. Autoantibodies to IFN‐λ1 and IFN‐λ2/3 are present in approximately 30% and 10% of APECED patients, respectively [141]. Anti‐T1 IFN autoantibodies can be detected early, even before the onset of clinical symptoms, and they serve as diagnostic and potentially prognostic markers for APECED [144, 145, 146]. Anti‐IFN‐α autoantibodies are highly neutralizing, recognize native conformational epitopes, and inhibit IFN‐stimulated gene expression [146, 147, 148]. Interestingly, the neutralizing capacity of these autoantibodies inversely correlates with the onset of T1D in APECED patients with GAD65 autoantibodies, suggesting a potential protective role against T1D and possible therapeutic applications [141]. Likely due to the neutralization of IFN‐α and IFN‐ω, some APECED patients experience severe manifestations of skin varicella‐zoster and mucosal herpes simplex infections [149].

The underlying cause of the development of autoantibodies against T1 IFNs remains unclear, mainly since these cytokines are not tissue‐specific and do not fit the conventional category of AIRE‐induced organ‐specific autoantigens. Importantly, T1 IFN autoantibodies are not exclusive to APECED but have been reported in approximately 70% of patients with thymoma [150, 151]. Thymomas originate from thymic epithelial cells and are more frequently associated with autoimmune diseases than any other tumor type [37]. Among these, myasthenia gravis is the most common, affecting 10%–50% of thymoma patients, depending on the histologic subtype. Interestingly, approximately 95% of thymomas lack AIRE expression [152]. Most thymomas have disorganized thymic microenvironment and produce a large population of polyclonal T cells, leading to the export of potentially autoreactive CD4+ T cells. The thymoma patients also develop autoantibodies to classical APECED autoantigens CYP21, CYP17, CYP1A1, TPH1, TH, AADC, GAD65, and NALP5, though typically at lower titers and frequencies than those observed in APECED patients [143].

Several pancreatic and steroidogenic autoantigens are expressed in the normal healthy thymus but not exclusively in the mTEC^hi^ population. In thymomas, the transcript levels of autoantigens that are frequently targeted by autoantibodies (CYP21, CYP17, TPH1, TH, TPO) did not correlate with AIRE expression [143]. This finding indicates that many key APECED autoantigens are AIRE‐independent despite their frequent recognition by autoantibodies in both APECED and thymoma patients. Thus, not all thymoma‐associated autoimmunity can be solely attributed to deficient autoantigen transcription in AIRE‐deficient thymic tumors. However, AIRE expression is correlated with thymic expression and genetic polymorphisms in the acetylcholine receptor (CHRA1), insulin, and thyroid‐stimulating hormone receptor (TSHR) genes, which encode proteins that are autoantigens in myasthenia gravis, type 1 diabetes (T1D), and Graves’ disease, respectively [23, 27, 153].

Considering the similarities of APECED and thymoma patients in the development of anti‐IFN‐α and disease‐associated pancreatic and steroidogenic AIRE‐independent autoantigens, a new model was proposed in which the main outcome of AIRE defect is active autoimmunization in parallel with negative selection of autoreactive clones [143, 150, 151]. In this model, AIRE typically contributes to the establishment of a tolerogenic environment in the thymic medulla. However, in its absence, and with the emergence of anti‐T1 IFN autoantibodies, the thymic environment shifts toward an autoimmunizing state, where autoantigens become targets of aberrant T cell activation. This concept of active thymic autoimmunization in APECED and thymomas parallels mechanisms observed in paraneoplastic autoimmune syndromes, where abnormal autoantigen expression triggers immune responses against self‐antigens. In this framework, AIRE‐deficient thymic environments become “dangerous,” fostering active autoimmunity rather than promoting tolerance.

In humans, autoantibodies targeting T1 IFN are found in other thymus‐related pathologies. They are present in IPEX, in patients with hypomorphic RAG1/RAG2 gene mutations and immunodeficiencies due to defects in NFKB2, NIK, RELB, CTLA4, IKZF2, and CD40L genes, of which several conditions are characterized by thymic disorganization and reduced expression of MHC class II and AIRE [154, 155, 156, 157]. Together, these findings emphasize the thymus's critical role in maintaining tolerance to T1 IFNs. Furthermore, studies conducted during the COVID‐19 pandemic identified a higher prevalence of T1 IFN autoantibodies in older individuals, exceeding 4% in those over the age of 70, possibly due to age‐related thymic decline [158]. These autoantibodies also increase susceptibility to severe COVID‐19 disease progression and adverse reactions to the yellow fever vaccine and West Nile virus encephalitis [158]. They also increase susceptibility to severe COVID‐19 disease progression and adverse reactions to the yellow fever vaccine and West Nile virus encephalitis [159, 160]. Also, neutralizing autoantibodies to T1 IFNs were found to increase the risk of life‐threatening COVID‐19 pneumonia in APECED patients [161].

## 10 Th17 Cytokines and Mucocutaneous Candidiasis

Although most prevalent against IFN‐α and IFN‐ω, the anti‐cytokine autoantibodies in APECED patients are not limited to T1 or T3 IFNs. Susceptibility to CMC in APECED is linked to autoantibodies against cytokines produced by Th17 cells. Like anti‐T1 IFN, the autoantibodies to Th17 cytokines emerge early in the disease course, with IL‐22 being the most commonly targeted (present in over 90% of patients). Autoantibodies to IL‐17F are found in approximately 75% of cases, and those against IL‐17A in 35%–50% [162, 163]. IL17 cytokine family consists of six members; IL‐17A, IL‐17B, IL‐17C, IL‐17D, IL‐17E and IL‐17F, and the patients do develop autoantibodies to only two members of the family. IL‐17A and IL‐17F, which are homologs and often act as heterodimers, are primarily produced by immune cells, including Th17 cells, ILC3, and γδ T cells. APECED patients exhibit impaired production of IL‐22 and IL‐17F by blood and skin T cells, and anti‐IL‐22 autoantibodies are detectable in saliva [162, 164]. Interestingly, some patients with CMC do not have detectable neutralizing autoantibodies, suggesting a broader dysfunction in Th17‐mediated immunity. In addition, autoantibodies to interleukins 1A, 5, 6, 10, and 18 have been reported in APECED [141, 144, 165].

## 11 Comparison Between APECED Patients and AIRE‐Deficient Mice

The species‐dependent differences in AIRE function have been noted before [166, 167]. Notably, mouse models do not fully recapitulate the human disease. Despite their well‐characterized defect in central tolerance, Aire‐deficient animals on C57Bl/6 or BALB/c background do not develop overt autoimmune disease, and tissue infiltrations are often mild or missing. Their life expectancy remains unchanged, and the function of infiltrated organs is generally unaffected, except for reduced fertility and blindness in a minority of cases. Among the two strains, BALB/c mice, which tend to exhibit a stronger humoral response, show more extensive tissue infiltration than C57Bl/6 mice. Curiously, Aire‐deficient mice develop a wasting disease when bred onto a non‐obese diabetic (NOD) background [168]. While NOD mice are a classical model for T1D and typically develop pancreatic islet infiltration (insulitis), Aire‐deficient NOD mice instead develop exocrine pancreatitis. In contrast, human patients rarely present with exocrine pancreatitis but often develop clinical T1D. Another autoimmune‐prone strain, SJL, also develops a wasting disease upon Aire gene knockout [168]. It appears that only autoimmune‐prone mouse strains with an Aire deficiency develop disease‐like features.

Aire mouse models do not develop the same profile of tissue‐specific autoantibodies as APECED patients [166]. Conversely, autoantigens identified in Aire‐deficient mice, such as interphotoreceptor retinoid‐binding protein (IRBP/RBP3) [25], odorant‐binding protein (OBP1a) [169], mucin 6 (MUC6) [170] or alpha‐fodrin (SPTAN1) [171] are not targeted in human APECED patients. Additionally, mouse models rarely develop infiltrations in the adrenal or parathyroid glands, which are typical targets in human disease. However, similarly to human patients, Aire‐deficient mice develop T cell responses and autoantibodies to melanoma‐specific cancer antigens, such as tyrosinase‐related protein (TYRP1/TRP1), which is expressed in mTECs in an Aire‐dependent manner [172, 173, 174].

In contrast to humans, the Aire‐deficient mice are not susceptible to oropharyngeal candidiasis [175]. Perhaps most strikingly, Aire‐deficient mice do not develop antibodies to T1 IFNs even at an older age [147]. However, in 1.5–2 year‐old Aire‐deficient mice on BALB/c (but not C57Bl/6) background, antibodies against IL‐17A have been detected, suggesting that aging and a stronger humoral response contribute to the development of anti‐cytokine autoantibodies [147]. Overall, these findings highlight substantial discrepancies in immune targets and disease pathogenesis between murine Aire‐deficient models and human APECED patients.

## 12 Comparison Between AIRE‐Deficient Mice and Rats

In contrast to AIRE‐deficient mice, AIRE‐deficient rats develop a more severe phenotype and exhibit overt autoimmune disease [176, 177]. This phenotype is evident in the outbred Sprague–Dawley strain, which is commonly used for its calm temperament and ease of handling. However, the disease is not specific to this strain, as AIRE knockout rats on inbred Brown Norway and Lewis backgrounds develop similar phenotypes. The similarity in disease presentation across different strains suggests that the genetic background in rats does not play a primary role in disease manifestation [176]. This feature distinguishes the rat model from the mouse models and more closely mirrors the human situation, where homozygous recessive AIRE mutations lead to disease manifestation regardless of genetic background.

AIRE‐deficient rats develop several clinical features resembling those seen in APECED patients, including alopecia, skin inflammation, depigmentation (resembling vitiligo), and nail dystrophy as features characteristic of ectodermal dystrophies. Lymphocytic infiltrations are widespread in multiple organs, with notable involvement of the salivary glands, pancreas, liver, and kidneys. Autoantibodies are produced against antigens in the kidney, liver, testes, intestines, adrenal glands, and pancreas. However, similar to AIRE‐deficient mice, but unlike human patients, the majority of AIRE‐deficient rats develop exocrine pancreatic infiltration rather than insulitis or T1D. Additionally, AIRE‐deficient rats do not develop overt chronic mucocutaneous candidiasis, the hallmark of human APECED.

Thymic development in AIRE‐deficient rats is comparable to that of wild‐type rats in terms of total thymocyte numbers and proportions. Similar to mouse models, these rats exhibit a reduction in thymic Foxp3+ Tregs and a downregulation of TRA expression in mTECs. Comparative transcriptomic analyses of TRA expression in the thymi of AIRE‐deficient rats and mice reveal that while AIRE regulates a similar set of self‐antigen genes across rodent species, certain distinctions exist that may account for species‐specific differences in autoimmunity. For instance, T1D‐associated insulin gene expression is downregulated in both species. In the periphery, the rat model also exhibits a decreased proportion of pDCs and activated NK cells.

A key distinction between the mouse and rat models is the development of autoantibodies against cytokines, particularly IFN‐α subsets. In AIRE‐deficient rats, anti‐IFN‐α autoantibodies are neutralizing, and their levels are comparable to those seen in human APECED patients. Nearly all affected rats develop high‐titer autoantibodies against IFN‐α by 7 months of age, with levels plateauing by 1 year. Additionally, rats develop autoantibodies to IL‐17A, though at a lower prevalence. Thus, the AIRE‐deficient rat model provides a valuable tool for studying the mechanisms driving the development of anti‐IFN‐α autoantibodies, their relationship with thymic expression, and their role in disease progression.

Recently, we demonstrated in a rat model that AIRE regulates the thymic expression of ISGs prior to the appearance of anti‐IFN‐α autoantibodies [178]. With the emergence of autoantibodies, the ISG levels were reduced in immune cells, along with lower NK cell activity. The presence of anti‐IFN‐α autoantibodies was associated with less tissue inflammation, suggesting they suppress T1 IFN signaling in AIRE‐deficient rat peripheral tissues. Overall, AIRE influences thymic IFN signaling in a manner that promotes the development of protective anti‐IFN‐α autoantibodies, which may help reduce autoimmune damage [178].

Although the Aire‐deficient rat model replicates many aspects of APECED syndrome, it also does not fully recapitulate the complete disease phenotype observed in human patients. This suggests that species‐specific immunological differences influence the manifestation of disease. Despite these limitations, the Aire‐deficient rat remains the most accurate preclinical model available for studying APECED.

## 13 Thymus as a Site of Tonic Expression of IFNs

Research on AIRE has predominantly relied on three investigative approaches: (i) Aire‐deficient mouse models, (ii) in vitro studies using overexpression of the AIRE gene, and (iii) analysis of immunological and pathological features of APECED patients. These studies have established a fundamental mechanism by which Aire promotes the transcription of a wide array of TRAs in mTECs, ensuring the proper negative selection of self‐reactive T cells and the differentiation of Tregs. However, the presence of high‐titer autoantibodies against T1 IFNs in nearly all APECED patients and the Aire‐deficient rat model introduces a paradox that challenges this well‐established model. It raises an intriguing question: is there an additional, yet unrecognized, aspect of thymic tolerance that involves T1 IFNs?

**FIGURE 1:**
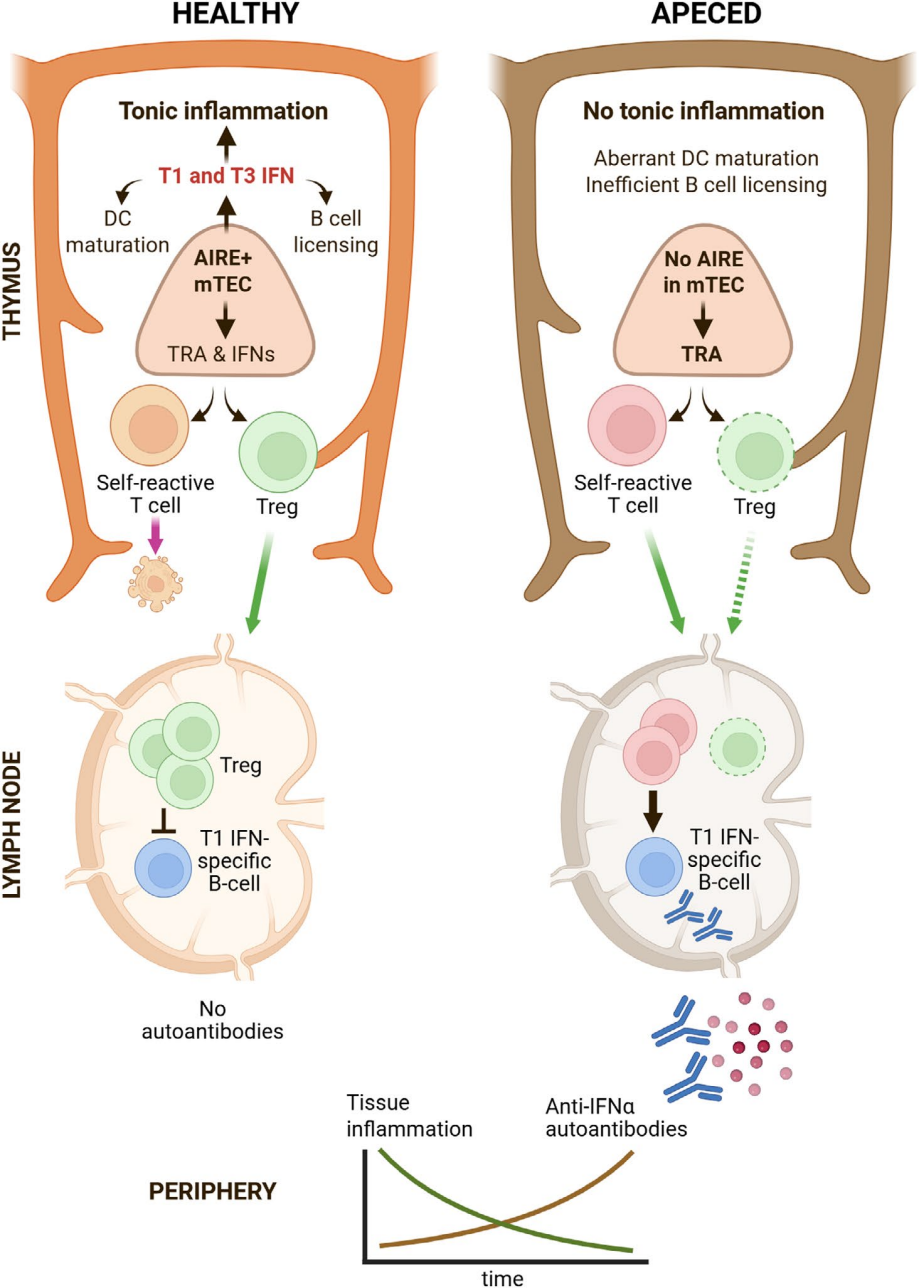
Model of development of autoantibodies to IFN‐α in AIRE‐deficient host. High‐level neutralizing autoantibodies against IFN‐α are detectable in the majority of APECED patients and in the Aire‐deficient rat model. The thymic expression of T1 and T3 IFNs is regulated by AIRE in mTEChi. In a healthy thymus, T1 and T3 IFN expression promotes tonic inflammation, which is essential for thymic DC maturation, B cell licensing, and the normal late‐stage differentiation of thymocytes. Moreover, central tolerance to interferons themselves is established in the thymus through Treg selection or clonal deletion. In the absence of AIRE, this tonic inflammatory environment is disrupted, leading to aberrant thymic function and impaired selection of autoreactive CD4^+^ T cells and Tregs. A deficiency in T1 IFN signaling results in a less diverse Treg repertoire, potentially reflecting the loss of specific Treg clones reactive to TRAs or ISGs. In contrast, impaired T3 IFN signaling leads to reduced numbers of thymic Tregs, likely due to its impact on DC1 maturation. This defective selection process allows IFN‐α‐specific T cells to escape central tolerance and activate B cells in secondary lymphoid organs, resulting in the production of anti‐IFN‐α autoantibodies. Remarkably, the presence of these autoantibodies correlates with reduced immune infiltration in the target tissues of Aire‐deficient animals, suggesting a protective role in mitigating autoimmune damage.

The development of T1 IFN autoantibodies in AIRE‐deficient hosts proposes a link between AIRE, T1 IFNs, and autoimmune pathogenesis (Figure 1). The shared disease severity and immunological features in human patients and Aire‐deficient rat models suggest that the presence of T1 IFN autoantibodies is not merely an epiphenomenon but a key contributor to disease progression. This is further supported by the association of T1 IFN autoantibodies with thymoma and other thymus‐related immune disorders in humans, suggesting that T1 IFNs play a crucial role in maintaining thymic immune homeostasis. Given this evidence, it is important to evaluate the role of T1 IFNs in thymic tolerance and determine whether their dysregulation contributes to the breakdown of self‐tolerance in APECED and related autoimmune disorders.

Tonic T1 IFN signaling appears to be a conserved feature of mammalian thymic architecture [179, 180]. Nevertheless, detecting T1 and T3 IFN expression at the mRNA and protein levels is particularly challenging due to their inherently low abundance and high biological sensitivity [181]. IFN‐α expression has been detected in the thymic medulla in humans, reinforcing the hypothesis that it plays a role in the thymus. Studies of healthy human thymus tissue have reported IFN‐α protein expression in CD68+ thymic macrophages and plasmacytoid dendritic cells (pDCs), enriched in the medullary region [144, 182, 183]. In murine models, pDCs were shown to produce IFN‐α, with significantly reduced expression observed in Aire‐deficient mice [184]. In contrast, an IFN‐β‐luciferase reporter mouse showed constitutive Aire‐dependent expression of the reporter gene in mTECs [185, 186]. A recent study by Ashby et al. analyzed Ifnβ1^tdTomato^ and Ifnλ2^eGFP^ mice for their reporter signals in the thymus and demonstrated Aire‐dependent IFN‐β and IFN‐λ expression in a small subset (~1%–2%) of Aire‐positive mTEC^hi^ cells [187]. The expression of these reporters followed the dynamics of Aire expression, indicating that Aire‐positive mTECs contribute to IFN‐β and IFN‐λ production, whereas hematopoietic cells were negative for reporter signals. Thymic myeloid cells were highly responsive to tonic expression of IFNs, impacting their maturation and activation [187]. Then, how to explain the findings of differential expression of T1IFN subsets IFN‐α and IFN‐β? One possible explanation for the differential expression of T1 IFN members in the thymus is that Aire‐driven tonic IFN‐β and IFN‐λ expression in mTECs expands IFN responsiveness in surrounding thymic myeloid cells, resulting in IFN‐α and ISG expression by pDCs and macrophages. It remains to be determined whether IFN‐α in the thymus is produced by different cell types from IFN‐β and IFN‐λ or whether this varies between species.

The mechanisms maintaining homeostatic expression of T1 or T3 IFN in Aire‐positive mTECs remain unknown. The IFN expression seems to be sterile, as it occurs independently of viral or pathogen‐related TLR signaling [187], and is not influenced by antigen exposure in an enriched environment [185]. The association of AIRE molecular function with double‐strand DNA breaks, Z‐DNA and other nonstandard DNA conformations raises an intriguing hypothesis of their role in T1 and T3 IFN production in mTECs as previous studies have demonstrated direct associations between the DNA damage response, cGAS‐STING pathway activation and T1 IFN production [188]. However, the IFN production in the thymus was neither dependent on the sensing of nonself or damage‐associated nucleic acids as mice deficient in both MAVS and STING1 did not show reduced expression of IFN gene‐driven reporters [187]. Thus, IFN production by mTECs is unlikely to be triggered by endogenous retroviruses or DNA breaks associated with AIRE‐mediated transcriptional activity. Further investigation into the role of AIRE nuclear bodies in this process is warranted, particularly given the functional parallels with PML bodies, which serve as regulatory hubs for IFN signaling [92].

Accumulating evidence suggests that low‐grade IFN expression is present and influences thymic function in multiple ways [187, 189, 190, 191]. The general T cell development seems not to be impaired in IFNAR1‐deficient mice; although, as expected, they exhibited decreased expression of IFN‐responsive genes Stat1 and Irf7 [180, 187]. However, in another study, the mice lacking IFNAR1 or STAT1 showed impaired deletion of autoreactive T cells in the male‐specific HY antigen model, along with an intrinsic inability of STAT1‐deficient thymocytes to undergo deletion by TCR‐mediated signaling [192]. The maturation of key thymic antigen‐presenting cells, the DC1 population, is regulated by IFN signaling [187]. Thymic DC1 participate in the selection of AIRE‐dependent Tregs by cross‐presenting TRAs acquired from mTEC^hi^ cells [30, 31], however, Tregs were reduced in Ifnlr1^−/−^ but not in Ifnar1^−/−^ mice, suggesting T3 IFN signaling important in Treg development. However, despite unchanged Treg numbers and DC maturation, the Treg TCR repertoire was less diverse in Ifnar1^−/−^ mice, suggesting an aberrant selection of TCRs, which may recognize ISG‐derived self‐antigens [187]. These findings suggested that thymic expression of T1 IFNs may have an important role in maintaining tolerance to ISG‐derived self‐antigens [187, 189]. However, in our analysis of APECED autoantibodies to over 9000 human proteins, we did not find ISGs to be enriched among positive autoantigen hits. Among almost 900 detected proteins, surprisingly few were TRAs, whereas many of them were associated with nucleic acid binding or proteins that are phosphorylated [142].

Thymic inflammation may not be limited to IFNs. Initiated by Aire‐positive mTECs expressing T1 and T3 IFNs, inflammatory signaling is likely amplified by other thymic cells, including dendritic cells, macrophages, and B cells. Furthermore, HCs in the medullary region may contribute to this function. Previous studies have shown that HCs produce thymic stromal lymphopoietin (TSLP), a cytokine that conditions dendritic cells to support Treg differentiation [193]. HCs have also been implicated in constitutive activation of thymic neutrophils and pDCs, promoting IFN‐α production. Proteomic analysis of the human thymic medulla identified post‐Aire cells and HCs as possessing a distinct pro‐inflammatory signature, including upregulation of the endogenous TLR4 agonist S100A8/S100A9. The contributions of T1 and T3 IFNs and other inflammatory signals by Aire‐positive mTECs and post‐Aire populations to thymic tolerance may provide a novel understanding of Aire function in the thymic immune tolerance beyond promiscuous gene expression.

## 14 Conclusion

The discovery of T1 IFN autoantibodies in APECED patients and Aire‐deficient rat model challenges the classical model of Aire‐mediated tolerance, necessitating a deeper exploration of T1 IFN function in the thymus. Evidence suggests that AIRE influences T1 and T3 IFN expression, shaping the inflammatory microenvironment required for thymocyte selection, Treg induction, and antigen presentation. However, the cellular outcomes of T1 and T3 IFN production by mTECs and their role in thymic tolerance remain incompletely understood. Further research is needed into these mechanisms, which will help to understand their potential role in thymic tolerance induction and reveal potential therapeutic ways to modulate thymic homeostasis.

## Funding

This work was supported by Estonian Research Competency Council (Grants PRG2011 and PRG377).

## Conflicts of Interest

The author declares no conflicts of interest.

## Data Availability

Data sharing not applicable to this article as no datasets were generated or analyzed during the current study.
